# Haplotypes on pig chromosome 3 distinguish metabolically healthy from unhealthy obese individuals

**DOI:** 10.1371/journal.pone.0178828

**Published:** 2017-06-01

**Authors:** Simona D. Frederiksen, Peter Karlskov-Mortensen, Sameer D. Pant, Maryse Guerin, Philippe Lesnik, Claus B. Jørgensen, Susanna Cirera, Camilla S. Bruun, Thomas Mark, Merete Fredholm

**Affiliations:** 1 Department of Veterinary and Animal Sciences, Faculty of Health and Medical Sciences, University of Copenhagen, Copenhagen, Denmark; 2 School of Animal and Veterinary Sciences, Charles Sturt University, Wagga Wagga, Australia; 3 INSERM UMR_S1166, Integrative Biology of Atherosclerosis Team, Paris, France; 4 Novo Nordisk, Scandinavia AB, Region Denmark, Maaloev, Denmark; Humboldt-Universitat zu Berlin, GERMANY

## Abstract

We have established a pig resource population specifically designed to elucidate the genetics involved in development of obesity and obesity related co-morbidities by crossing the obesity prone Göttingen Minipig breed with two lean production pig breeds. In this study we have performed genome wide association (GWA) to identify loci with effect on blood lipid levels. The most significantly associated single nucleotide polymorphisms (SNPs) were used for linkage disequilibrium (LD) and haplotype analyses. Three separate haploblocks which influence the ratio between high density lipoprotein cholesterol and total cholesterol (HDL-C/CT), triglycerides (TG) and low density lipoprotein cholesterol (LDL-C) levels respectively were identified on Sus Scrofa chromosome 3 (SSC3). Large additive genetic effects were found for the HDL-C/CT and LDL-C haplotypes. Haplotypes segregating from Göttingen Minipigs were shown to impose a positive effect on blood lipid levels. Thus, the genetic profile of the Göttingen Minipig breed seems to support a phenotype comparable to the metabolic healthy obese (MHO) phenotype in humans.

## Introduction

Obesity is defined as excessive accumulation of fat in the body to the extent that it may have a negative effect on health. According to the World Health Organization (WHO), in 2016 obesity had more than doubled worldwide since 1980. Obesity can be socially stigmatizing, however, in itself it is not the primary health problem. Rather, it is the co-morbidities such as cardiovascular disease (CVD) and type 2 diabetes (T2D) which pose the major health problems. Common to many of the co-morbidities is an unhealthy metabolic profile with insulin resistance and dyslipidemia characterized by elevated triglycerides (TG) and low-density lipoprotein cholesterol (LDL-C) levels and by decreased high-density lipoprotein cholesterol (HDL-C) concentrations [1]. Approximately 25% of obese humans do, however, not present the metabolic complications and do not suffer from an increased susceptibility towards obesity related diseases [2, 3]. These individuals are characterized as 'metabolically healthy obese' (MHO) [4].

To understand why MHO individuals have a better prognosis than the metabolically unhealthy individuals it is relevant to elucidate the genetic mechanisms underlying regulation of blood lipids such as cholesterol and TG.

Cholesterol is a ubiquitous steroid and a vital component of cellular membranes in vertebrates. But at the same time, subendothelial accumulation of cholesterol is the cause of atherosclerotic lesions leading to vascular diseases, heart attacks, aortic aneurysms and peripheral vascular diseases [5] which, altogether, represent the most frequent causes of demise in the industrialized world [6].

Cholesterol is transported in plasma in lipoprotein particles. These consist of a major structural apolipoprotein, peripheral apolipoproteins, structural lipids (phospholipids and cholesterol) and a cargo of TG and steryl esters. Dietary lipids are absorbed via the small intestine and packaged as TG into large particles known as chylomicrons (CM) which distribute lipids throughout the body for direct use and storage. CM remnants return to the liver where the remaining lipid cargo enters hepatic lipid pathways and a second class of lipoprotein particles is produced; the very low density lipoproteins (VLDL). Like CM, VLDL distributes TG to the periphery. As VLDL delivers TG they shrink and shed peripheral apolipoproteins, hence the proportion of cholesterol increases and VLDL becomes low-density lipoproteins (LDL). Both VLDL and LDL are characterized by the major structural apolipoprotein APOB. LDL can bind to cells expressing the LDL-receptor (LDLR) and as such, LDL acts as an efficient cholesterol delivery system into cells [5].

The reverse transport of cholesterol from the periphery to the liver is mediated by high-density lipoproteins (HDL) containing the major structural apolipoprotein APOA1. Nascent HDL particles contain few lipids but collect free cholesterol and phospholipids from peripheral tissues. Different mechanisms enrich the HDL particles with lipids, which ultimately are delivered to the liver via Scavenger Receptor B1 (SCARB1) for subsequent excretion with the bile [5].

Heritability for LDL-C and HDL-C levels in humans are estimated to be around 70%.[7] Large-scale genome wide association studies (GWAS) in humans have discovered over 150 common genetic variants associated with plasma lipids [8]. However, these loci only explain a small fraction of the total variance in blood lipids [9] and the bulk of genetic factors for dyslipidemia are still unaccounted for.

A way to elucidate novel mechanisms involved in blood lipid regulation is to study animal models. Animal models provide the benefits of a strictly controlled diet and environment which is impossible in large human studies. Consequently, random noise is greatly reduced and a corresponding increase in power is presumed when animal models are used to study human conditions. Also, the individual breeds are genetically more homogeneous than humans due to domestication and artificial selection [e.g. ref. 10]. GWAS [11] and quantitative trait locus (QTL) mapping [12] have been performed in mice to identify loci in the genome with effect on blood lipids. But more frequently, spontaneous dyslipidemic and genetically engineered mouse models have been used to study the effect of specific dyslipidemia associated genes first identified in human GWAS studies [13]. However, market differences in metabolism and adipose tissue biology exists between rodents and humans [e.g. ref. 14].

The pig is an animal model with a close similarity to humans in body size, physiology, organ development and disease progression. It has been widely used as a model for cardiovascular and metabolic diseases [15]. The presence of atherosclerotic lesions in aorta was described in pigs as early as 1954 [16]. The close similarity to vascular lesions in human atherosclerosis have later been confirmed [17, 18], and the pig has proved its value as a model for this disease [19].

In the present study, a GWAS aimed at identifying loci with effect on blood lipid levels is reported. The GWAS was performed in two pig crosses established by using Göttingen Minipig as the parental boar line in both crosses whereas Duroc and Yorkshire production pigs were used as parental sow lines in the two crosses respectively. The Göttingen Minipig is an obesity prone pig breed [20] often used in studies of obesity, diabetes and metabolic syndrome [21–24], whereas Duroc and Yorkshire pigs have been bred for leanness for decades. Haplotypes were defined around the most significantly associated single nucleotide polymorphisms (SNPs), and the effect of the haplotypes with the highest additive effects were studied further.

## Materials and methods

### Experimental animal model, sample collection and blood lipid analysis

Two populations were produced as F2 crosses using purebred Göttingen Minipig (M), Duroc (D) and Yorkshire (Y) as parental lines (Ellegaard Göttingen Minipigs A/S, Dalmose, Denmark; DanBred International, Herlev, Denmark). The Minipig-Duroc (MD) crossbred F1 animals were founded by seven M boars and seven D sows and 28 F1 MD gilts and 16 F1 MD boars were used to produce 285 F2 animals. Similarly, the Minipig-Yorkshire (MY) crossbred F1 animals were founded by seven M boars and seven Y sows and 279 F2 animals were produced by 26 MY gilts and 13 MY boars (for further information see [25, 26]).

All pigs were raised under controlled environmental conditions and fed the same diet ad libitum. The project was approved by the Danish Animal Experiments Inspectorate. Animal care and maintenance were conducted according to the Danish “Act about Animal Husbandry” and “Animal Protection Act” (Act 432, July 9, 2004; Act 1150, Sep. 12, 2015). All pigs were housed at a regular pig farm, and slaughtered at a commercial slaughterhouse by stunning and bleeding under veterinary supervision.

Blood samples for blood lipid analysis were drawn from the jugular vein at about two month of age (63 ± 10 days, abbreviated Age1) and at slaughter (242 ± 48 days, abbreviated Age2). Plasma lipid levels were assayed by standardized techniques using a Konelab 20 Clinical Chemistry Analyzer (Thermo Scientific, Sweden) and commercial reagent kits from Roche Diagnostics for Total Cholesterol (CT) and from ThermoElectron for TG and HDL-C levels (direct method). LDL-C levels were calculated using the Friedewald formula. Observations >5 SD from the mean were considered outliers and excluded. Box-Cox transformation was used to adjust for non-normality. After transformation, skewness and kurtosis were calculated for each phenotypic distribution and Q-Q plots were made to evaluate normality.

### Estimation of genetic parameters

Genetic parameters were estimated using Best Linear Unbiased Prediction (BLUP) based on Average-Restricted Maximum Likelihood (AI-REML) using DMU version 6, release 5.2 [27]. Variance components were estimated using two different univariate models depending on whether the phenotypes were measured at Age1 (Model 1) or Age2 (Model 2):

$${1)\;y}_{i}=\mu +{\mathrm{SEX}}_{i}+{\mathrm{CROSS}}_{i}+{\gamma (\mathrm{AGE}}_{i})+\xi A_{i}+\varepsilon_{i}$$

$${2)\;y}_{i}=\mu +{\mathrm{SEX}}_{i}+{\mathrm{CROSS}}_{i}+\gamma_{1}{(\mathrm{AGE}}_{i})+\gamma_{2}{{(\mathrm{AGE}}_{i})}^{2}+\xi A_{i}+\varepsilon_{i}$$

where y is the phenotype for animal i, μ is the population mean, SEX and CROSS are fixed effects for sex and cross (MD or MY) for animal i, AGE for animal i is a covariate with regression coefficient γ and A is the additive genetic effect for animal i with the regression coefficient ξ. εi is the residual error. A and ε are assumed to be independent and normally distributed with variances Aσ_ANIMAL_^2^ and Iσ_ε_^2^, respectively. A is the additive genetic relationship matrix based on pedigree information, σ_ANIMAL_^2^ the additive genetic variance, I an identity matrix of appropriate size and σ_ε_^2^ the residual error variance.

A bivariate linear mixed model was fitted to estimate phenotypic and genetic correlations between pairs of traits (Trait 1 and Trait 2):

$$\left[\begin{array}{c} y_{1}\\ y_{2} \end{array}\right]=\left[\begin{array}{c} X_{1}\\ 0 \end{array}\begin{array}{c} 0\\ X_{2} \end{array}\right]\left[\begin{array}{c} b_{1}\\ b_{2} \end{array}\right]+\left[\begin{array}{c} Z_{1}\\ 0 \end{array}\begin{array}{c} 0\\ Z_{2} \end{array}\right]\left[\begin{array}{c} a_{1}\\ a_{2} \end{array}\right]+\left[\begin{array}{c} e_{1}\\ e_{2} \end{array}\right]$$

where y_1_ and y_2_ are vectors of the transformed phenotypic measurements of Trait 1 and Trait 2 respectively. X_1_ and X_2_ are design matrices connecting the measurements to b_1_ and b_2_ which are vectors of the environmental fixed effects, while Z_1_ and Z_2_ are design matrices linking the measurements to a_1_ and a_2_ which are vectors for the random genetic effects for each trait. Finally, e_1_ and e_2_ are vectors of the random environmental effects. The effects fitted in the bivariate linear mixed models are the same as the ones fitted in the univariate linear mixed models. When fitting the bivariate model, the non-diagonals of the additive genetic variance and covariance matrix G for animal effects and the variance and covariance matrix R for random residual effects were included as follows:

$$A\sigma_{A}^{2}=G=\left[\begin{array}{c} A\sigma_{\mathrm{A1}}^{2}\\ A{\mathrm{cov}}_{A21} \end{array}\begin{array}{c} A{\mathrm{cov}}_{A12}\\ A\sigma_{A2}^{2} \end{array}\right]\;I\sigma_{e}^{2}=R=\left[\begin{array}{c} I\sigma_{E1}^{2}\\ I{\mathrm{cov}}_{E21} \end{array}\begin{array}{c} I{\mathrm{cov}}_{E12}\\ I\sigma_{E2}^{2} \end{array}\right]$$

where cov_A12_ = cov_A21_ is the additive genetic covariance and cov_E12_ = cov_E21_ the random residual covariance between Trait 1 and 2. The phenotypic covariance cov_P12_ = cov_P21_ between Trait 1 and 2 can be calculated as: cov_P12_ = cov_A12_ + cov_E12_ [28].

### Genotyping and Quality Control

Each animal was genotyped using the 60k porcine Illumina SNPchip. Sex chromosomes were excluded from the single-marker analyses. The Sus Scrofa 10.2 pig genome assembly was used to derive map positions for all SNPs. After genotyping, quality control (QC) was conducted using GenABEL version 1.8–0 [29] excluding: animals and SNPs with more than 5% missing genotypes; SNPs with a minor allele frequency (MAF) less than 5%; SNPs that significantly deviated from Hardy-Weinberg equilibrium (HWE) at a significance threshold of p < 1E-05. After QC, 549 pigs and 44,554 SNPs remained.

### GWAS using single-marker test and estimation of SNP effects

GWAS was carried out for each phenotype using a single-marker test as implemented in GenABEL [30]. The analyses were performed in two steps: In step 1, the polygenic linear mixed models were defined as Model 1 (for Age1) and Model 2 (for Age2) where A_i_ is the random additive polygenic effect for animal i based on identity by state (IBS). A_i_ is assumed to be normally distributed with a (co)variance of **G**σ_ANIMAL_^2^ where **G** is the genomic relationship matrix and σ_ANIMAL_^2^ the additive polygenic variance. Estimation of effects was performed by means of maximum likelihood (ML) [31].

Step 2 use estimated residuals from step 1 (contains part of the QTL variance) to estimate SNP effects [30]:

$${\hat{\varepsilon }}_{i}=\mu +SNP_{m}+\varepsilon_{m}$$

where ${\hat{\varepsilon }}_{i}$ is the estimated residual error from step 1, μ is the mean, SNP_m_ is assumed a fixed effect with 3 genotype scores 0, 1 and 2 referring to the number of minor allele copies for the m’th SNP and ε_m_ is the residual error. Each single SNP was modeled independently. The allele substitution effect of the m’th SNP was calculated as the average phenotypic change when replacing a major allele with the minor allele [32].

SimpleM [33–35] was used to correct for multiple testing by calculating the effective number of independent tests M_eff_ = 12916. Missing genotypic values were replaced with the common allele genotype in the genotype matrix prior to the analysis. It resulted in a cut off value of–log10(0.05/12916) = 5.41 corresponding to a nominal significance level (α) of 3.87*10^−6^.

For each significant SNP, the phenotypic variance explained by the SNP was calculated as [36]:

$$V_{\mathrm{SNP}}=V_{m}=2q_{m}(1-q_{m})S\hat{N}P_{m}{}^{2}$$

where V_m_ is the phenotypic variance explained by the m’th SNP, q_m_ is MAF for the m’th SNP and ${S\hat{N}P}_{m}$ is the estimated effect for the m’th SNP. The variance explained by the SNP is assumed to be due to additive genetic variance. The proportion of additive genetic variance explained by each SNP was calculated as V_SNP_ / σ_A_^2^. The additive genetic effect (α) for each top-SNP was calculated using BLUP as implemented in DMU where the effect of having 0, 1 or 2 copies of a specific SNP was fitted in the models described above (Model 1 and Model 2).

### Linkage disequilibrium and haplotype analysis

The method described by Gabriel et al.(2002) [37] as implemented in Haploview [38] was used to define haploblocks around the top SNPs identified in the GWAS. Default parameters were used except for “Fraction of strong LD in informative comparisons” which was set to >0.80 (default >0.95). Phased haplotypes for each haploblock for each individual were estimated using the—hap-phase option in Plink [39]. The additive genetic effect of each haplotype was estimated using BLUP by fitting the haplotype as a covariate in the models described above (Model 1 and Model 2). Effect were fitted for one haplotype variant at a time by computing the effect of having 0, 1 or 2 copies of that variant. Effects were only calculated for haplotype variants observed in the founder animals and not for variants found in less than 20 animals in the F2 population.

To evaluate haplotype effects in individuals with high- and low-BMI respectively, all F2 animals were sorted according to BMI and the one-third with highest BMI (high BMI) and lowest (low BMI) respectively were selected. Each of the groups were subdivided according to haplotypes in the HDL-C/CT and the LDL-C QTL regions and mean and standard deviation for BMI_Age2, CT_Age2, TG_Age2, HDL-C/CT_Age1, HDL-C_Age2, HDL-C/CT_Age2, LDL-C_Age2 and LDL-C/HDL-C at Age2 were calculated separately using Students-t test for animals which were homozygous for either the GM or the Yorkshire/Duroc haplotypes in the HDL-C/CT and LDL-C QTL regions.

## Results

A moderate genetic correlation was found between Age1 and Age2 both in regard to LDL-C and HDL-C/CT, whereas, for TG there was no correlation between measurements at the two ages (see Table 1).

**Table 1:** Genetic (rA) and phenotypic (rP) correlation between phenotypes measured at different age.

Phenotypes		Correlation coefficients	
Trait 1	Trait 2	r_A_ (SE)	r_P_
TG_Age1	TG_Age2	0.01 (0.28)	-0.07
LDL-C_Age1	LDL-C_Age2	0.41 (0.19)	0.31
HDL-C/CT_Age1	HDL-C/CT_Age2	0.21 (0.24)	0.29

Age1: 63 ± 10 days, Age1; Age2: 242 ± 48 days

The GWA study resulted in the identification of several QTL regions below the nominal significance level on *sus scrofa* chromosome 3 (SSC3) (see Table 2). A single SNP on SSC3 at 124.7 Mb is associated with TG_Age2, whereas, there is no indication for an association with TG_Age1 at this position (data not shown). Conversely, the same loci on SSC3 are associated with LDL-C at Age 1 and Age 2 and the same is true for HDL-C/CT. The most significant associations for LDL-C are found around 125.6 Mb and the most significant SNPs for HDL-C/CT are found at 122.8 Mb.

**Table 2:** QTL regions and SNP effects.

Phenotypes	Most significant SNP on SSC3									SNP effect
	SNP name	Position	P-value	MA	MAF	MAF (GM)	MAF (DD)	MAF (YY)	V_SNP_/σ_A_^2^	α (SE)
TG_Age2	ALGA0021201	124739382	3.35E-06	A	0.36	0.00	0.70	0.83	0.34	-0.02 (0.00)
LDL-C_Age1	ASGA0094490	125568713	1.34E-08	A	0.38	0.00	0.60	0.83	0.17	0.05 (0.01)
LDL-C_Age2	ASGA0106214	125592465	2.07E-07	A	0.20	0.41	0.00	0.00	0.13	-0.05 (0.01)
HDL-C/CT_Age1	ALGA0021148	122811986	2.12E-09	G	0.39	0.00	0.60	1.00	0.20	-0.04 (0.00)
HDL-C/CT_Age2	MARC0019977	122854537	3.91E-07	A	0.42	0.00	0.70	1.00	0.26	-0.03 (0.01)

MA: Minor allele; MAF: Minor allele frequency; MAF (GM), (DD), (YY): Minor allele frequency in the parental generation of Göttingen minipigs, Duroc and Yorkshire respectively; VSNP/σA2: Proportion of additive genetic variance explained by each SNP; α (SE): additive genetic effect for each top-SNP (standard error)

**Fig 1:**
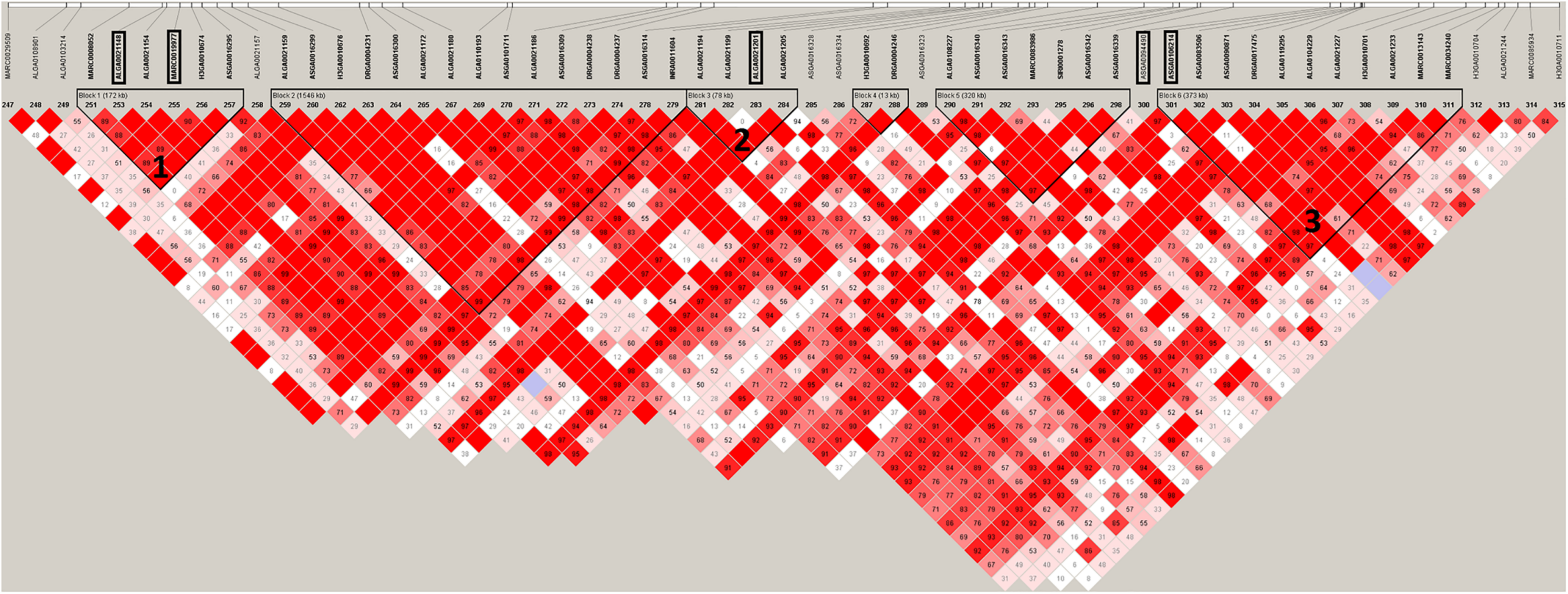
LD in a 2.8 Mb region (122.4–126.1 Mb) on Sus Scrofa chromosome 3. Frames mark top SNPs for HDL-C/CT_Age1, HDL-C/CT_Age2, TG_Age2, LDL-C_Age1 and LDL-C_Age2, in this order from left to right. Triangles indicate haploblock structure in the QTL region. Block 1: QTL for HDL-C/CT; Block 2: QTL for TG; Block 3: QTL for LDL-C.

LD analysis (Fig 1) revealed that the two top SNPs for HDL-C/CT_Age1 and HDL-C/CT_Age2 are located close together and belong to the same LD-block defined by six SNPs from 122.7–122.9 Mb (designated '1' in Fig 1). The top SNP’s for TG_Age2 and LDL-C_Age1 and Age2 show minimal LD to surrounding SNPs and are not included in any LD blocks when default parameters in Haploview are used (results not shown). Relaxing the parameters for block definition, as described above, includes the top SNP for LDL-C_Age2 into an LD block defined by 10 SNPs spanning a region from 125.6–125.9 Mb (designated '3' Fig 1), but the top SNP for LDL-C_Age1 is still outside the LD block. In the same way the relaxed parameters include the top SNP for TG_Age2 into a four SNP haploblock from124.67–124.75 Mb (designated '2' in Fig 1) clearly separated from the HDL-C/CT and the LDL-C loci. The SNPs located in the haploblocks associated with the lipid levels are listed in SI 1.

The additive genetic effect of haplotypes present in more than 20 animals is shown in Table 3. The largest additive effects appear for the HDL-C/CT and LDL-C haplotypes for which there is also a clear breed specific segregation. For the HDL-C/CT associated haplotypes one specific haplotype, AAAGGG, impose a higher HDL-C/CT level. Twelve out of fourteen Göttingen Minipig founders are homozygous for this haplotype and the remaining two has one copy of AAAGGG. The haplotype GGGAAA, for which all Yorkshire founders are homozygous, has an equivalent negative effect, that is, animals with this haplotype have lower HDL-C/CT levels. The same trend is true for the haplotypes segregating from the Duroc breed, and among the Duroc haplotypes, AGAAGA, has an even stronger negative effect compared to the Yorkshire haplotype on the HDL-C/CT level. The effect of the individual haplotypes is similar at Age 1 and Age 2 (data not shown).

**Table 3:** Haplotype effects—SSC3.

Phenotype	Haplotype	Haploblock	Additive effect	SE	Origine*
**HDL-C/CT**	AAAGAA	1	-0.02	0.01	D
	AAAGGA		0.01	0.01	GM
	AAAGGG		0.04	0.00	GM
	AGAAGA		-0.07	0.01	D
	AGGAAA		-0.02	0.02	D
	GAAAGA		-0.02	0.02	D
	GGGAAA		-0.04	0.01	Y
**TG Age2**	AAAA	2	-0.02	0.00	Y, D
	GACA		0.01	0.00	GM, Y
	GGAA		-0.02	0.01	D
	GGCG		0.01	0.01	D
**LDL-C**	AGGAGCCAAA	3	-0.04	0.01	GM
	GAAAGAAACG		0.01	0.01	D
	GGAAGAAACG		-0.02	0.02	Y
	GGAAGAAGAG		0.03	0.02	Y
	GGAAGAAGCG		0.02	0.01	Y
	GGGAGCCAAA		0.01	0.01	GM
	GGGAGCCGAG		0.00	0.02	D
	GGGGAACAAG		0.02	0.01	D

Effect of haplotypes present in more than 20 animals in the population in the three QTL haploblocks

*: GM = Göttingen Minipig; Y = Yorkshire; D = Duroc

For the LDL-C associated LD block, the haplotype AGGAGCCAAA originating from the Göttingen Minipig founders, decrease the LDL-C level. The Yorkshire founder specific haplotypes, GGAAGAAGAG and GGAAGAAGCG, increase LDL-C level; however, there is also one Yorkshire haplotype, GGAAGAAACG, which decreases LDL-C level. Two of the Duroc haplotypes increase the LDL-C level slightly. The effect of each haplotype is the same at Age 1 and Age 2 (data not shown) even though the blocks are only loosely in LD according to the LD analysis.

To further evaluate the phenotypic consequences of the haplotypes segregating from Göttingen Minipig and Yorkshire/Duroc respectively we compared lipid and TG levels in animals homozygous for either Göttingen Minipig haplotypes or the Duroc/Yorkshire haplotypes. The comparison was performed in the one-third of the F2 animals with the highest and one-third with lowest BMI, respectively. The mean BMI in the combined F2 population (564 animals) is 127±20 while in the selected high BMI group (136 animals) the mean BMI is 154±13, and in the selected low BMI group (132 animals) the mean BMI is 111±9. As seen in Table 4, within the low BMI group of animals there are no differences in TG and lipid values between the genetically diverse animals. However, when comparing animals homozygous for Göttingen Minipig haplotypes with animals homozygous for Yorksire/Duroc haplotypes within the high BMI group of animals both TG and lipid values (except HDL-C) are significantly higher in animals with the Yorkshire/Duroc haplotypes.

**Table 4:** Effects of HDL-C/CT and LDL-C haplotypes with high additive effect in high- and low-BMI individuals.

	BMI_Age2	CT_Age2	TG_Age2	HDL-C/CT Age1	HDL-C_Age2	HDL-C/CT Age2	LDL-C_Age2	LDL-C/HDL-C Age2	n
**High BMI**									
**GM/GM**	157.56 (14.00)	2.28 (0.49)	0.45 (0.18)	0.54 (0.07)	1.40 (0.31)	0.62 (0.07)	0.68 (0.27)	0.49 (0.17)	28
**D/D or Y/Y**	149.75 (16.05)	2.55 (0.55)	0.71 (0.35)	0.47 (0.06)	1.30 (0.35)	0.51 (0.10)	0.93 (0.33)	0.79 (0.46)	22
**t-test**	0.037468	0.034815	0.000744	0.00044	0.164139	7.38E-05	0.002637	0.001605	
**Low BMI**									
**GM/GM**	112.62 (9.38)	2.26 (0.84)	0.44 (0.23)	0.51 (0.10)	1.25 (0.54)	0.56 (0.10)	0.80 (0.35)	0.68 (0.31)	32
**D/D or Y/Y**	112.81 (6.00)	2.28 (0.67)	0.46 (0.17)	0.45 (0.07)	1.28 (0.44)	0.57 (0.11)	0.79 (0.39)	0.67 (0.41)	22
**t-test**	0.465747	0.464812	0.420131	0.017532	0.420304	0.379878	0.44152	0.462115	

Mean and standard deviation (in parentheses) for eight phenotypes in high and low BMI animals homozygous for the Göttingen Minipig haplotypes (GM/GM) or Yorkshire (Y/Y) or Duroc (D/D) haplotypes in haploblock 1 (HDL-C/CT) and 3 (LDL-C).

## Discussion

In agreement with previously performed GWA studies in pigs [26, 40, 41], we have identified a QTL region influencing lipoprotein traits on SSC3. The homologous region on human chromosome 2 has also been implicated in the regulation of blood lipids in human GWA studies [42]. Our study shows that three separate haplopblocks influence HDL-C/CT -, TG -, and LDL-C levels respectively (see Fig 1). The HDL-C/CT and HDL-C QTL loci flank the TG_Age2 locus and thus, this 2.8 Mb region on SSC3 seems to encompass several loci with a regulatory effect on different plasma lipids. This is underpinned by the observation that the SSC3 locus affects HDL-C and LDL-C levels independent of age but for TG-levels there is only an association to TG_Age2 and no evidence suggesting an association to TG_Age1. This indicates that different genes or regulatory mechanisms in the region are responsible for effects on cholesterol and triglyceride levels, respectively. This is also in agreement with the observed correlations between phenotypes at different ages (Table 1) and the lack of a correlation between TG at Age1 and Age2.

There are no annotated known genes in the HDL-C/CT and TG associated SSC3 haploblocks in the Sscrofa 10.2 assembly but the gene encoding ras homolog family member B (*RHOB*) is located in the LDL-C associated haploblock. RHOB is a member of the RHO GTP-binding protein family which regulates expression of *CD36* [43]. CD36 is a scavenger protein with high affinity for plasma lipoproteins including LDL and a high expression in tissues such as skeletal muscle, heart, mammary, epithelium and adipose tissue, with a very active fatty acid metabolism [44]. The gene encoding apolipoprotein B (*APOB*) is located at 125.23–125.35 Mb. It is an obvious candidate gene for lipid related traits but in the present study it is not included in associated LD-blocks even though it was covered by four SNPs with a MAF above the exclusion threshold. The gene encoding lipid droplet associated hydrolase (*LDAH*) is located at 125.42–125.53 Mb which is also outside the identified LD-blocks. Further studies including sequencing and genotyping of additional markers are warranted to ascertain LD structure and to identify genetic variation. It is very likely that the observed differences in blood lipids are caused by variation in regulatory components within the identified QTL regions in particular since a high number of different genes involved in regulation of lipoproteins are located within this region of the genome.

Investigation of the additive genetic effect of the most common haplotypes segregating within the F2 population indicates that the haplotypes originating from the Göttingen Minipig breed provide a healthier lipid profile compared to the haplotypes segregating from the Yorkshire and Duroc breeds. I.e., Göttingen Minipig haplotypes in haploblock 1 appears to increase HDL-C/CT compared to the Yorkshire and Duroc haplotypes, which have the opposite effect. Conversely, in haploblock 3 the Göttingen Minipig haplotype lowers the level of LDL-C and the Yorkshire and Duroc haplotype increases LDL-C level with the exception of the GGAAGAAACG haplotype segregating from the Yorkshire breed. This effect is confirmed by comparing animals homozygous for the Göttingen Minipig and Duroc/Yorkshire haplotypes respectively within groups of animals with high and low BMI respectively. Within the high BMI group animals with Göttingen Minipig haplotypes have a significantly lower TG level, lower levels of LDL-C, and LDL-C/HDL-C ratio and higher ratio of HDL-C to CT (Table 4). Within the low BMI group there is no difference in the phenotypes between the two genetic variants. Thus, overall Göttingen Minipig seems to have a genotype that supports a more healthy blood lipid profile in spite of the fact that they are prone to obesity. Or conversely, pigs without the Göttingen Minipig haplotypes develop a more unhealthy, dyslipidemic profile together with obesity, compared to pigs with Göttingen Minipig haplotypes which uphold a healthy lipid profile despite development of severe obesity.

The Gottingen Minipig breed was developed in the 1960's using Minnesota Minipigs, Vietnamese Potbelly Pigs, and German Landrace as founders (Simianer and Köhn, 2010). A likely explanation for the MHO profile in Göttingen Minipigs is that the obesity prone minipig founders have been adapted to overcome obesity by natural selection. Thus, unexpectedly, the results presented here indicate that the Göttingen Minipig breed is not well suited for studies of the obesity related co-morbidities but may be a valuable model to advance understanding of the MHO phenotype in humans.

The MHO profile identified in our F2 population is comparable to the MHO profile in humans which appear to be protected against obesity related metabolic complications. However, although MHO is an important, emerging phenotype in humans no universally accepted definition has been established for this phenotype yet [45]. It is also debated to what extend MHO individuals will remain healthy [46, 47]. On the other hand, studying MHO subjects may lead to better intervention strategies for metabolically unhealthy obese people, and elucidate if they by lifestyle changes or by the use of medicine can switch to a better metabolic profile.

In conclusion, we have substantiated that different genetic loci have an effect on TG early and late in life. We have also substantiated that the genomic region close to genes implicated in lipoprotein metabolism (*RHOB*, *APOB*, *LDAH*) comprise regulatory elements of importance to the regulation of lipid metabolism. Interestingly, the largest additive genetic effects of the haplotypes identified in this study show that haplotypes segregating from the obesity prone Göttingen Minipig breed is able to uphold a healthy lipid profile despite development of obesity. Thus, the genetic profile of the Göttinging Minipig breed seems to support a phenotype comparable to the MHO phenotype in humans promoting this pig breed as a model for further studies of this particular phenotype. Our future studies will be directed at identification the genetic variation in the regulatory components involved in lipid metabolism and further genetic characterization of the healthy metabolic phenotype.
